# Modeling person guessing as a random effect: a Bayesian approach of the two-parameter logistic model

**DOI:** 10.3389/fpsyg.2026.1678086

**Published:** 2026-02-16

**Authors:** Georgios Sideridis, Mohammed Alghamdi

**Affiliations:** 1 Boston Children’s Hospital, Harvard Medical School, Boston, MA, United States; 2 Department of Self-Development Skills, King Saud University, Riyadh, Saudi Arabia

**Keywords:** 2PLE model, aberrant responding, Bayesian estimation, guessing behavior, person-level guessing, test security, test-taking behavior

## Abstract

**Introduction:**

Guessing behavior has been an enduring problem that undermines the validity and interpretability of scores from MC items. The present study implements a Bayesian random-effects extension of the 2PLE model which suggests that guessing is a latent individual trait rather than a single item parameter.

**Methods:**

We implemented a Monte Carlo simulation in a fully crossed design of sample sizes (*N* = 100–1,000) and test lengths (6–40 items), with 50 replications per condition. Item response data were simulated under the 2PLE model with heterogeneous guessing.

**Results:**

In all conditions the estimates of discrimination were larger with the 2PLE than with the 3PL. Gains were especially marked for item difficulty and lower-asymptote estimation that had noticeable distortion under the incorrect 3PL model. Bayesian predictive fit indices (i.e., Leave-One-Out Information Criterion, LOOIC; Widely Applicable Information Criterion, WAIC) consistently supported the 2PLE model under all sample sizes and test lengths. In the proposed framework, the person-level random effect δ_*n*_ reflects differences between individuals in guessing tendency and directly influences the lower asymptote of an item response function.

**Discussion:**

Through reallocating guessing variance from items to persons the 2PLE random-effects model can better capture diversified response patterns, and obtain a better psychometric performance. Findings are consistent with the conceptualization of guessing as a substantive trait-based process and underscore the utility and necessity of using person-specific guessing models to optimize inferences from test scores.

## Introduction

The reliability and validity of test scores have always been essential for standardized educational and psychological measurement. One of the eminent threats to these properties according to broad-based (multifaceted) test development models is guessing in multiple-choice response formats because it compromises the construct relevance of measurement error associated with correct responses from guessing versus indifference rather than aptitude *per se* (Downing and Haladyna, 2004; Wise and DeMars, 2006). Consequently, the guessing behavior, random, educated, strategic, implies that interpretations of scores can also be distorted and the validity or reliability of test results may be compromised. Empirical evidence has indicated increased awareness that guessing can threaten test validity of constructs assessed by standardized tests, and alternative assessment and scoring procedures have been advocated to reduce its influence (e.g., Lee and Bolt, 2018). This study has broader implications for the field of educational and psychological measurement by providing an alternative analytical model which can accurately model guessing as a person attribute.

### Psychometric issues and conceptual underpinnings of person guessing behavior

Guessing places construct-irrelevant error variance into test scores (Downing and Haladyna, 2004) and biases Item Characteristic Curves (ICCs) in Item Response Theory (IRT). The 3PL model includes a *pseudo-guessing* (*c*) that represents the lower asymptote of the item characteristic curve, i.e., the probability of a correct response for very low-ability examinees who may still benefit from partial knowledge or elimination of distractors (Birnbaum, 1968). This approach may capture the statistical deference of guessing but may not fully account for motivational or cognitive factors. This is explicated by Kane and Moloney (1978) who model the answer-until-correct (AUC) method. Their use of a modified Horst model suggests that item reliability under AUC is highly sensitive to examinees’ abilities to eliminate wrong choices, and the number of distractors included in the test. Guessing has, thus, the greatest negative impact on item and scale reliability.

In the measurement literature, several distinct forms of guessing have been discussed, each reflecting different cognitive or motivational processes. Random guessing refers to indiscriminate responding in the absence of item-specific knowledge, typically yielding chance-level accuracy. Lucky guessing denotes correct responses obtained by chance despite lack of knowledge and is therefore an outcome rather than a behavioral strategy. Rapid guessing describes a time-based disengagement behavior in which examinees respond quickly without attempting item solution (Wise and Kong, 2005 In contrast, ability-based guessing, as defined by the 2PLE model of Zhu et al. (2019), assumes that the probability of successful guessing when guessing happens is a deterministic function of ability, that is, examinees with the same ability have the same chance to guess right. We extend this model to incorporate person-based guessing, which we conceptualize as a latent trait reflecting individual differences in the tendency to guess (δ_*n*_) systematically and nonrandomly across content beyond ability. This proposition gives expression to the idea that individuals at the same ability level under guessing may have different baseline probability of correct responding, corresponding to stable motivational or strategic differences rather than random noise.

## Model description: moving from the 3PL to the 2PLE and 2PLE random model

The item response function of the 3PL IRT model is given by Equation 1:


$$P\,\left(\theta \right)=c_{i}+\left(1-c_{i}\right)\cdot \frac{1}{1+\exp\,\left(-a_{i}\,\left(\theta -b_{i}\right)\right)},$$ (1)

Where *P*(θ) is the probability of a correct response given an ability level θ, *a_i_* is the item discrimination parameter, *b_i_* is the item difficulty parameter, and *c_i_* is the lower asymptote representing the guessing effect at the item level (Birnbaum, 1968). In the 3PL model (Birnbaum, 1968), the lower asymptote *c_i_* is treated as an item-specific fixed parameter and is typically estimated across all examinees under the assumption that the pseudo-guessing parameter *c* is an item-specific constant shared across examinees. Although its effect on the probability of a correct response is modulated by ability via the logistic term, the 3PL does not allow individual differences in guessing tendencies beyond what is implied by θ itself. While this formulation is computationally convenient, it fails to account for known inter-individual variability in guessing propensities due to differences in motivation, strategy use, or test-taking behavior (Wise and Kong, 2005).

In contrast, the 2PLE model proposed by Zhu et al. (2019) reconceptualized guessing as a person–item interaction, with a logistic function governing the pseudo-guessing as a function of person ability. The model incorporates a latent trait-conditioned guessing function that depends not solely on item-level characteristics but also on person-specific parameters, thereby allowing individual differences in guessing behavior to be explicitly modeled and quantified (for the multidimensional case see Youxiang et al., 2024). The 2PLE model introduced by Zhu, Wang, and Tao conceptualized guessing as a deterministic function of the person’s ability. Specifically, the guessing probability *g*_*ni*_ was defined as Equation 2:


$$g_{\mathrm{ni}}^{\mathrm{Zhu}}=\gamma +\left(1-\gamma \right)\cdot \frac{1}{1+\mathrm{ex}\,\left(-a_{\text{i}}\,\left(\theta_{\text{n}}-b_{\text{i}}\right)\right)}$$ (2)

In the above equation, γ is 1/k for k-options items (e.g., 0.25 for a 4-choice item). This baseline guessing formulation is specific to the original 2PLE model and is not retained in the present extension. In other words, in the Zhu et al model, there is no individual-specific guessing parameter; rather, for examinees who do guess, the *conditional* probability of a correct response increases with θ, reflecting the assumption that more able examinees can more effectively eliminate implausible options and thus guess more *accurately* (Zhu et al., 2019). This does not imply that high-ability examinees guess more often, only that their guesses, when they occur, are more likely to be correct. There is no individual-specific pseudo-guessing—rather, all persons with the same θ level have the same guessing probability. In other words, successful guessing is entirely governed by the levels of the latent trait θ and the properties of the discrimination and difficulty parameters *a_i_*, and *b_i_*. The Zhu et al. model introduced a dynamic, ability-based lower asymptote, in an effort to correct of potential shortcoming of the 3PL model, which treats guessing behavior as an item-level constant. In Zhu et al.’s (2019) model, guessing is entirely determined by ability with guessing being modeled at the person level. Thus, individuals with the same θ are predicted to have identical levels of successful guessing. In the present model we attempted to add a layer of individuality and variability in guessing for individuals with the same θ.

To visualize the substantive impact of the person-level guessing trait, ICCs for a representative item are plotted at low, medium, and high values of δ_*n*_, illustrating how δ_*n*_ shifts the lower asymptote while preserving the ability-based slope. As δ_*n*_ increases, the person-level lower asymptote *g*_*n*_ increases, producing a systematically elevated baseline probability of success among low-ability examinees. This visualization makes explicit how the 2PLE random-effects formulation accommodates heterogeneity in guessing behavior that the 3PL model cannot represent.

### Psychological Interpretation of the *δ*_n_ trait

The latent person parameter δ_*n*_ is conceptualized as an individual difference reflecting the examinee’s propensity to engage in guessing. Psychologically, δ_*n*_ may reflects a latent propensity toward non-construct-relevant responding, which may arise from disengagement, impulsivity, strategy use, or motivational factors. Modeling δ_*n*_ allows the response function to capture systematic deviations from construct-relevant behavior and helps disentangle whether low-ability examinees are performing poorly because of limited skill or because of erratic or disengaged response patterns. In this way, δ_*n*_ plays a role analogous to person-fit indices in classical test theory but is integrated directly into the IRT response model. Importantly, δ_*n*_ is not intended to represent unsystematic error or residual variance, but rather a latent behavioral tendency inferred probabilistically from response patterns, reflecting systematic individual differences in engagement and response strategy that are orthogonal to ability.

### Current model specification and estimation: the 2PLE with random person guessing

The item response function of the proposed 2PLE model with random person guessing for person *n* on item *i* is modeled via Equation 3:


$$P\left(Y_{n\,i}=1|\theta_{n},a_{i},b_{i}\right)=g_{n\,i}+\left(1-g_{n\,i}\right)\cdot \frac{1}{1+\exp\,\left(-a_{i}\,\left(\theta_{n}-b_{i}\right)\right)}$$ (3)

where θ_*n*_ denotes the latent ability of person *n_i_* and *a*_*i*_,*b*_*i*_ are the item discrimination and difficulty parameters, respectively. In contrast to traditional IRT models such as the 3PL, which employ a fixed itemlevel pseudo-guessing *c_i_*, the proposed model introduces a person-specific guessing probability *g*_*ni*_, reflecting interindividual variability in test-taking behavior.

The probability of guessing is modeled as Equation 4:


$$g_{n}={\mathrm{logit}}^{-1}\,\left(\delta_{m}\right)$$ (4)

Where $\delta_{n}∼𝒩\,\left(0,\sigma_{\delta }^{2}\right)$ is a person-specific latent trait. The behavior of this formulation lies on influencing the lower asymptote, moving it in upward or downward fashion depending on individual’s proclivity to guess. The logistic function ensures that less able and higher-delta-responders have higher probabilities of guessing correctly (which is in line with whether these are disengaged or strategic responders). In this study, the lower asymptote at baseline was fixed as zero so that person- specific guessing parameter δ_*n*_ would range over the full [0, u] range instead of being raised from an artificially high starting point from 1/k in multiple choice testing as used in Zhu’s et al., original 2PLE model. By treating guessing as a latent individual-difference variable, the proposed model offers a more nuanced account of test-taking behavior as more than just aberrant responding.

**FIGURE 1:**
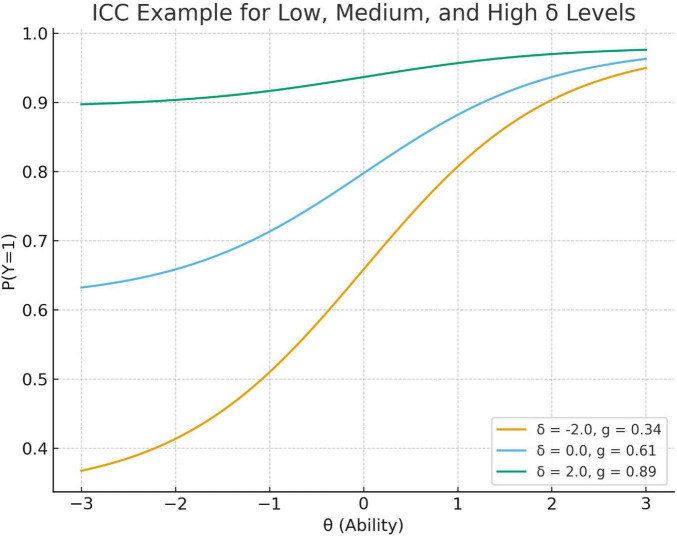
Item characteristic curves (ICCs) for a single item under low/medium/high values of δ_n_. Item characteristic curves (ICCs) for a representative item (*a* = 1.2, *b* = 0) under low (δ_n_ = - 2.0), medium (δ_n_ = 0), and high (δ_n_ = 2.0) values of the person-specific guessing trait in the 2PLE random-effects model. Higher δ_n_ values shift the person-conditional lower asymptote upward, particularly at low ability levels, reflecting an increased probability of correct responses attributable to guessing. As ability increases, ICCs converge because the ability-based logistic component dominates the response probability. The figure illustrates how δ_n_ introduces interindividual heterogeneity in guessing behavior beyond what is captured by ability alone.

In more detail the present model extends the framework of Zhu et al. (2019) by incorporating a random effect for person-level pseudo-guessing that accounts for variability among individuals in guessing. Thus, the 2PLE model assumes an ability-dependent deterministic guessing function of item-condensing guessing based on person-independent item-specific guessing (which is modulated by δn), while the present generalization in turn introduces a random latent variable δ(n) permitting systematic dispersion of guessing behavior across persons other than what can be predicted from classic IRT formulations such as the 3PL. By including factor δn, the model separates guessing from θ and permits better estimation of both ability and item parameters. This partition is a way to reduce construct-irrelevant variance which would otherwise be attributed either to θn (inflating ability estimates) or to ai (deflating discrimination) (Figure 1).

In conclusion, the model we proposed extends the dynamic person-item interaction framework of 2PLE by introducing person-specific random effect to the lower asymptote. This extension permits heterogeneity in guessing behavior for different examinees, even given ability. Functionally, the model (a) accommodates variance in test-taking behavior that is unrelated to trait ability, (b) enhances parameter estimation by accounting for systematic guessing bias, and (c) affords new person-level diagnostics: examinees with high or low δn can be flagged as “high guessers” or “low guessers,” respectively. By conceptualizing guessing as a latent individual difference variable, PP and Ppb provide a better understanding of test-taking behavior in terms of underlying motivational, cognitive, or affective tendencies and support the identification and interpretation of abnormal response patterns.

### Computation of item guessing using person-based estimates

Following estimation, we computed average person-based guessing probability estimates at the item level i as follows Equation 5:


$${\bar{g}}_{i}=\frac{1}{N}\,\sum_{n=1}^{N}g_{\mathrm{ni}},$$ (5)

where *g*_*ni*_ denotes the estimated guessing probability for person *n* on item *i*, calculated from the posterior medians of the person and item parameters-namely *a*_*i*_,*b*_*i*_,θ_*n*_, and δ_*n*_. These item-level averages, ${\hat{g}}_{i}$, reflect a descriptive summary and not an estimated parameter of the lower asymptote of each item’s item characteristic curve (ICC), but unlike the fixed pseudo-guessing *c_i_* in the 3PL model, they reflect a dynamic interaction between person traits and item properties.

### Implications for measurement and practice

Estimating person- level guessing tendencies has several substantive advantages over the traditional approach of examining guessing at the item level. The first is that identification of examinees showing unusually high guessing behavior can help make inferences about the validity of a test, as well as the effort or strategy of the examinee. Second, it purifies ability estimates, particularly for low ability examinees who may respond in non-construct-relevant ways. Third, it strengthens the item-level diagnostics, the overall average guessing rate on an item level would give context sensitive information on item working under realistic testing conditions.

### Analytical choice: opting for the Bayesian model

From a technical standpoint, Bayesian estimation offers distinct advantages over maximum likelihood (ML) methods when modeling item response theory (IRT) models that incorporate person-level guessing behavior. In traditional ML estimation, parameters such as person abilities θ_*n*_, and item characteristics-including discrimination *a_i_*, difficulty *b_i_*, and possibly guessing *c_i_*-are estimated by maximizing the likelihood function *L*(θ,*a*,*b*,*c*∣*Y*), where *Y* is the observed item response matrix. However, such point estimates can be unstable in the presence of guessing-related behaviors, which often flatten or distort the likelihood surface and lead to convergence difficulties or local maxima. Furthermore, ML estimation relies on asymptotic approximations to quantify uncertainty, which may be invalid in complex models or with sparse data. By contrast, Bayesian methods rely on full posterior inference (Equation 6):


p(|θ,α,b,c|Y)∝p(|Y|θ,α,b,c)⋅p(θ,a,b,c), (6)

where *p*(θ,*a*,*b*,*c*) denotes prior beliefs about parameters. This approach inherently carries the uncertainty through the model and gives full posterior distributions for all parameters, leading to more reliable conclusions. According to Dienes (2014), Bayesian statistics can evaluate competing hypotheses, such as ability-based processing vs. deviant guessing. Bayesian estimation also characterizes parameter identifiability problems via the use of informative or weakly informative priors. For example, restricting pseudo-guessing to a plausible range (e.g., [0.2, 0.3] for four-point multiple-choice items) enhances stability, particularly when data are corrupt with noise or careless responding. Such regularization is crucial to accurately model systematic variation in guessing behavior across individuals, as in person-fit analyses, or random person-level pseudo-guessing models. In conclusion, the Bayesian estimation provides a principled and flexible approach to modeling complex response behaviors in IRT formulations. It enhances parameter recovery, allows for model-based uncertainty, and facilitates form-fit comparisons using criteria such as LOOIC or Bayes factors. These benefits are particularly crucial in models that have the goal to separate ability from irrelevant variance (e.g., strategic guessing or disengagement scenarios) for which ML approaches are often not suitable. In the 3PL comparison model, we employed an identical Bayesian MCMC framework again with weakly informative priors on c to keep it within plausible values [e.g., c ∼ Beta(α, β) centered around 1/k for k-option items], which follows standard practice in Bayesian estimation of 3PL pseudo-guessing parameters.

Research on test security has proposed a broad suite of statistical and algorithmic approaches to detecting outlier behavior, including similarity indices (Wollack, 1997), person-fit indices like lz and Ht (Cizek and Wollack, 2017; van der Linden and Sotaridona, 2006), divergence-based measures based on Kullback–Leibler or Jensen–Shannon distances (Fox and Marianti, 2016; Lin, 1991; Sinharay, 2016). These methods target behaviors such as: Answer copying, pre-knowledge, random responding and lucky guessing. While informative, these methods operate diagnostically after the response pattern is generated. The current model complements this literature by embedding aberrant tendencies directly into the response function through the δ_*n*_ parameter, thus integrating aberrance detection and ability estimation within a single framework. Whereas traditional test security indices aim to flag aberrant response patterns after test administration, the proposed 2PLE framework probabilistically attributes guessing-related aberrance during model estimation, thereby integrating detection and correction within a single psychometric model

A related literature investigates rapid guessing using response times (Schnipke and Scrams, 2002; Wise and Kong, 2005). Such models distinguish solution behavior from disengaged responding based on speed, but do not directly model the probability of success conditional on guessing. The present approach is complementary: whereas response-time models infer disengagement behaviorally, the 2PLE framework models guessing probabilistically within the item response function. This provides a psychometrically principled method for representing individual differences in guessing even in the absence of response-time data.

Empirical evaluations of response-time–based rapid-guessing methods generally support their utility for identifying low-effort responding, especially in low-stakes settings; however, their effectiveness depends on the availability and quality of response-time data and on defensible thresholding or mixture assumptions that can vary across tests, devices, and examinee subgroups. Moreover, response-time approaches primarily classify effort states (e.g., solution vs. rapid responding) rather than estimating a person-level propensity that directly parameterizes the probability of a correct response under guessing. Consequently, even when response times are available, they do not provide a fully probabilistic decomposition of observed accuracy into construct-relevant ability versus guessing-related success. The present 2PLE random-effects approach addresses this gap by modeling heterogeneity in guessing success directly in the item response function, enabling identification and adjustment for person-level guessing tendencies even in datasets where response times are unavailable.

The primary research question guiding this study is whether modeling guessing as a person-level latent trait improves psychometric performance relative to the traditional Three-Parameter Logistic (3PL) model when guessing behavior varies across individuals. Specifically, we examine whether a Two-Parameter Logistic Extension with random person-level guessing (2PLE) yields superior parameter recovery, reduced bias, and improved predictive model fit compared to the 3PL model. To address this question, we employ a Monte Carlo simulation design in which response data are generated under a person-level guessing mechanism and analyzed using both models. Model performance is evaluated using parameter recovery indices and Bayesian measures of predictive fit, including LOOIC and WAIC. The following section describes the simulation design, data generation procedures, estimation approach, and evaluation criteria in detail.

## Materials and methods

### Simulation design

The simulation study was conducted according to Monte Carlo procedures (de Ayala, 2009; Muthén and Muthén, 2002), where multiple datasets are generated under known true conditions to check parameter recovery and model fit. Sample sizes (*N* = 100–1,000) and test lengths (*I* = 6–40) were chosen based on educational/psychological testing ranges typically encountered in empirical studies (Lord, 1980; de Ayala, 2009). Fifty replications per condition were selected, given that previous Monte Carlo research has shown this number is sufficient for estimating validly parameter estimates, thus, being computationally feasible. Person-level guessing propensities δ_*n*_ were generated from normal distributions with varying standard deviations (σδ) and transformed to the unit interval via the logistic function to obtain valid guessing probabilities *g*_*n*_ ∈ (0,1). This transformation ensures probabilistic coherence while allowing unbounded latent heterogeneity on the δ_*n*_ scale. Two values of σδ (0.5 and 0.8) were selected to represent moderate and moderately high guessing heterogeneity; additional values are examined in sensitivity analyses reported in Supplementary Appendix.

### Data generation

The present study adopts a Monte Carlo simulation design in which dichotomous item responses are generated under a two-parameter logistic extension model with random person-level guessing (2PLE random-effects model). This design allows direct evaluation of parameter recovery and model fit when guessing behavior varies systematically across individuals.

#### Generating person-level parameters

For each replication and simulation condition, person abilities were drawn independently from a standard normal distribution (Equation 7),


$$\theta_{n}∼𝒩\,\left(0,1\right),n=1,\ldots ,N,$$ (7)

reflecting the conventional assumption of normally distributed latent ability in unidimensional IRT models. In addition, each person was assigned a latent guessing propensity (Equation 8),


$$\delta_{n}∼𝒩\,\left(0,\sigma_{\delta }^{2}\right),$$ (8)

where σ_δ_ controls the degree of heterogeneity in individual guessing behavior. In the present study, σ_δ_ = 0.8 was selected to represent a moderate-to-high level of interindividual variability in guessing propensity, consistent with empirical findings on disengaged and aberrant responding in low-stakes and operational testing contexts. Person-specific guessing probabilities were obtained via a logistic transformation (Equation 9):


$$\rho_{n}={\mathrm{logit}}^{-1}\,\left(\delta_{n}\right),$$ (9)

which maps the unbounded latent trait δ_*n*_ onto the unit interval (0,1), yielding valid lower-asymptote probabilities. In the present implementation, the baseline lower bound γ was fixed at 0, such that all lower-asymptote variation is captured by the person-specific parameter ϑ_*n*_. Across simulation conditions, the implied distribution of *g_n_* exhibited substantial heterogeneity, with values spanning nearly the full unit interval (see Table 1 for specifications of generated parameters).

**TABLE 1:** Generating parameter distributions and approximate ranges used in the simulation.

Parameter	Generating distribution	Mean	SD	Approximate range (min-max)	Notes/justification
*θ*_n_	Normal (0, 1)	0	1	≈ [−3, 3]	Standard latent ability distribution
δ_n_	Normal (0, 1)	0	σδ∈{0.5, 0.8}	≈ [−3, 3] (varies by σδ)	Person-level guessing propensity; varied in sensitivity analyses
*g*_n_	Logit^−1^ (δ_*n*_)	≈ 0.50	≈ 0.15	≈ [0.05, 0.95]	Implied person-specific lower asymptote; nonlinear transform of δ_*n*_
*a*_i_	Lognormal (log μa, A_sd)	≈ 1.15	≈ 0.25–0.35	≈ [0.3, 3.5]	Item discrimination; correlated with *b_i_* (ρ = −0.30)
*b*_i_	Normal (0, 1)	0	1	≈ [−3, 3]	Item difficulty distribution
U	Constant	0.98	—	Fixed	Upper asymptote reflecting near-perfect performance ceiling
Corr (*a*_i_, *b*_i_)	—	−0.30	—	—	Empirical evidence

Approximate ranges correspond to the central 99% of the generating distributions. Values of *g*_n_ reflect the implied distribution after logistic transformation of δ_n_.

#### Generating item-level parameters

Item discrimination and difficulty parameters were generated jointly to reflect realistic psychometric conditions observed in applied testing. Specifically, item parameters (*a*_*i*_,*b*_*i*_) were drawn from a correlated bivariate distribution (Equation 10),


$$\log\,\left(a_{i}\right),b_{i}∼{𝒩}_{2}\,\left(\left(\begin{array}{c} \text{log}\,\left(1.0\right)\\ 0 \end{array}\right),\left(\begin{array}{cc} 0.30^{2}\,\rho_{a\,b} & \\ \rho_{a\,b}\,1.0^{2} & \end{array}\right)\right)$$ (10)

with the correlation ρ_*ab*_ = −0.30, reflecting empirical evidence that more discriminating items tend to be moderately easier in operational assessments. Discrimination parameters were exponentiated to ensure that non-negative values are present, resulting in a lognormal distribution for *a_i_*, while difficulty parameters followed a normal distribution centered around zero.

#### Response probability model

Using person and item parameters, the probability of a correct response for individual *n* on item *i* was computed in line with the 2PLE random-effects model as follows Equation 11:


$$P\left(Y_{n\,i}=1\right)=\rho_{n}+\left(u-\rho_{n}\right)\cdot \mathrm{logit}\left(\alpha_{i}\left(\theta_{n}-b_{i}\right)\right),$$ (11)

where *g_n_* reflects the person-specific lower asymptote and *u* the upper asymptote. In the present study, we fixed the upper asymptote at *u* = 0.98, allowing some minor variability from perfect performance.

Dichotomous item responses were generated from Equation 12:


$$Y_{n\,i}∼\mathrm{Bernoulli}\left(P\left(Y_{n\,i}=1\right)\right)$$ (12)

With individual differences in guessing behavior directly contributing to the baseline probability of success, especially at low-levels of theta, while preserving the standard logistic relationship between ability and item difficulty at higher levels of theta. For each simulation condition, summary statistics of the generated person- and item-level parameters are shown in Table 1.

### Model estimation procedure

All simulated datasets were analyzed within a Bayesian framework using Stan via the CmdStanR interface. Models were estimated using Hamiltonian Monte Carlo with the No-U-Turn Sampler (NUTS) as implemented in CmdStan (version 2.36.0) and CmdStanR (version 0.8.0), executed in R (version 4.3.x). For each dataset, four Markov chain Monte Carlo (MCMC) chains were run with 2,000 iterations per chain, including 1,000 warm-up iterations and 1,000 post-warm-up sampling iterations (see Supplementary Appendix 1 for Stan code).

Person-level latent parameters for ability (*θ*_*n*_) and guessing propensity (δ_*n*_) were estimated using a non-centered parameterization, whereby standardized latent variables were drawn from standard normal distributions and subsequently transformed via location and scale parameters. This parameterization was adopted to improve sampling efficiency and reduce posterior correlations between latent effects and their variance components. Item parameters were estimated using centered parameterizations with weakly informative priors.

Weakly informative priors were specified using the same functional families as the data-generating distributions but with hyperparameters chosen to span a broad range of plausible values rather than to closely reproduce the generating mechanism. Specifically, item discrimination parameters were assigned lognormal priors, item difficulty parameters normal priors, person abilities normal priors, and person-level guessing propensities normal priors. During estimation, item and person parameters were assigned independent priors, even when they were generated from correlated distributions, to avoid artificially favoring the data-generating model.

Convergence was assessed using multiple diagnostic criteria. Across all simulation conditions, the Gelman–Rubin statistic (R̂) was below 1.01 for all parameters, and both bulk and tail effective sample sizes exceeded 400. No divergent transitions were observed. In addition, trace plots for representative parameters were visually inspected to confirm stable mixing. Posterior predictive checks further indicated that the fitted models adequately reproduced key characteristics of the simulated data.

For comparability, the same MCMC settings, priors, and convergence criteria were applied to the Bayesian 3PL model. In the 3PL specification, guessing is modeled as an item-level fixed parameter rather than a person-level latent trait and therefore does not involve a hierarchical variance component.

Posterior samples were used to compute the pointwise log-likelihood of observed responses, which served as the basis for Bayesian model comparison. Two widely used measures of out-of-sample predictive accuracy were calculated: (a) the Leave-One-Out Information Criterion (LOOIC) using Pareto-smoothed importance sampling, and (b) the Widely Applicable Information Criterion (WAIC). Both indices were computed using the loo package in R. For each simulation condition, fit statistics were aggregated across the 50 replications to obtain mean and standard deviation estimates of LOOIC and WAIC, providing stable summaries of model performance across varying sample sizes and test lengths.

#### Prior predictive checks

Prior predictive simulations were conducted to ensure that the chosen priors yielded plausible item response probabilities and asymptote behavior. The person-specific guessing probability was defined as ϑ_*n*_ = logit^−1^ (δ_*n*_), and the response probability followed Equation 13:


$$P\left(\gamma_{n\,i}=1\right)=q_{n}+\left(u-q_{n}\right)\cdot \left(logit^{-1}\left(\omega_{i}\left(\theta_{n}-b_{i}\right)\right)\right).$$ (13)

Prior predictive checks confirmed that the implied distributions of *q_n_* remained within psychometrically reasonable ranges.

### Evaluation criteria

For each condition and parameter type (θ,σ,*b*,*c*/g), we computed (Equation 14):


$$\mathrm{RMSE}=\sqrt{\frac{1}{R}\,\sum_{r=1}^{R}{\left({\hat{\psi }}_{r}-\psi \right)}^{2}},$$ (14)

where ${\hat{\psi }}_{r}$ is the mean posterior estimate in replication *r*,ψ is the true value, and *R* = 50 is the number of replications. Bias was defined as Equation 15:


$$\mathrm{Bias}=\frac{1}{R}\,\sum_{r=1}^{R}\left({\hat{\psi }}_{r}-\psi \right).$$ (15)

Coverage was the proportion of replications in which the 95% posterior credible interval for ψ contained the true value. Global model fit was assessed using the leave-one-out information criterion (LOOIC) and the widely applicable information criterion (WAIC), computed from the pointwise log-likelihoods using the loo package; lower values indicate better out-of-sample predictive performance.

### Model comparison

We computed PSIS-LOO–based stacking weights and pseudo-BMA weights (see Supplementary Appendices 2–4).

## Results

A Monte Carlo simulation was conducted to compare the performance of the Three-Parameter Logistic (3PL) model with the Two-Parameter Logistic model incorporating a latent person-specific guessing component (2PLE). The simulation design crossed six sample sizes (*N* = 100, 150, 200, 300, 500, 1,000) with five test lengths (*I* = 6, 10, 20, 30, 40), yielding 30 unique conditions. For each condition, 50 replications were generated and evaluated with respect to parameter recovery and predictive performance. Evaluation criteria included root mean square error (RMSE), bias, and coverage for all parameters, as well as two Bayesian model-fit indices: the Leave-One-Out Information Criterion (LOOIC) and the Widely Applicable Information Criterion (WAIC). MCMC diagnostics demonstrated stable estimation across all conditions, with $\hat{R}=1.01$, adequate effective sample sizes, and zero divergent transitions (see Supplementary Appendix 2).

### Parameter recovery

For all the considered sample sizes and test lengths, the 2PLE model results in lower RMSE and absolute bias than those obtained for all parameters (θ, a, b and guessing) with the 3PL model. Coverage rates for the 2PLE model were close to nominal in all but one condition (see Supplementary Appendix 2), whereas the 3PL showed systematic under coverage of difficulty and guessing parameters as a result of model misspecification. Importantly, no reversals were observed across the N × test-length design, indicating that the superiority of the 2PLE model is stable rather than condition-specific.

### Item discrimination (a)

**FIGURE 2:**
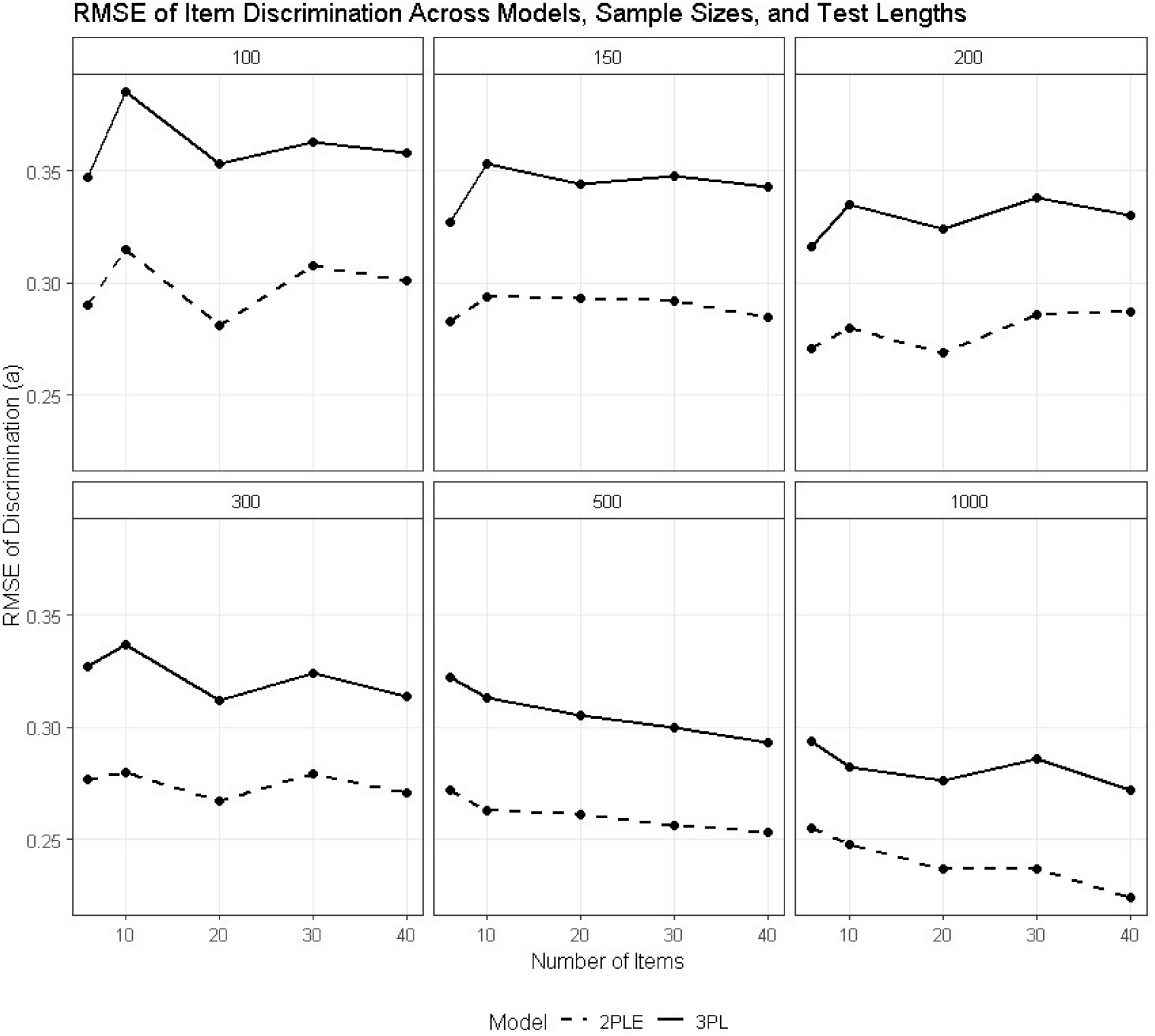
RMSE estimates for 3PL and 2PLE random models across simulation conditions for the discrimination parameter.

Across all sample sizes and test lengths, the 2PLE model consistently demonstrated superior recovery of item discrimination parameters relative to the 3PL model (Table 2). RMSE values for *a_i_* were uniformly lower under the 2PLE model. For the smallest design condition (*N* = 100, I = 6), RMSE for discrimination decreased from 0.347 under the 3PL to 0.290 under the 2PLE model. This pattern was more pronounced with increasing sample size and test length; at *N* = 500 and I = 6, RMSE values were 0.322 for the 3PL and 0.272 for the 2PLE, respectively, and similar differences were observed across lengthier tests (Figure 2).

**TABLE 2:** Comparative performance of 3PL and 2PLE random models on RMSE.

Sample size	Item count	RMSE-θ-3PL	RMSE-θ-2PLE R	RMSE-a-3PL	RMSE-a-2PLE R	RMSE-b-3PL	RMSE-b-2PLE R	RMSE-c-3PL	RMSE-g-2PLE R
100	6	0.928	0.901	0.347	0.290	1.442	0.437	0.425	0.167
150	6	0.922	0.899	0.327	0.283	1.421	0.378	0.425	0.165
200	6	0.935	0.911	0.316	0.271	1.441	0.333	0.422	0.163
300	6	0.928	0.906	0.327	0.277	1.433	0.308	0.424	0.165
500	6	0.932	0.910	0.322	0.272	1.446	0.274	0.424	0.164
1,000	6	0.932	0.912	0.294	0.255	1.417	0.224	0.424	0.164
100	10	0.899	0.871	0.385	0.315	1.467	0.476	0.428	0.160
150	10	0.912	0.878	0.353	0.294	1.452	0.379	0.426	0.159
200	10	0.904	0.877	0.335	0.280	1.446	0.345	0.423	0.160
300	10	0.894	0.859	0.337	0.280	1.428	0.295	0.424	0.159
500	10	0.897	0.869	0.313	0.263	1.421	0.248	0.423	0.159
1,000	10	0.905	0.871	0.282	0.248	1.431	0.213	0.424	0.159
100	20	0.857	0.814	0.353	0.281	1.393	0.452	0.423	0.148
150	20	0.855	0.811	0.344	0.293	1.416	0.387	0.428	0.147
200	20	0.857	0.814	0.324	0.269	1.401	0.356	0.424	0.150
300	20	0.866	0.819	0.312	0.267	1.418	0.301	0.424	0.150
500	20	0.863	0.815	0.305	0.261	1.419	0.272	0.426	0.148
1,000	20	0.866	0.812	0.276	0.237	1.442	0.216	0.424	0.148
100	30	0.841	0.773	0.363	0.308	1.340	0.441	0.424	0.142
150	30	0.831	0.771	0.348	0.292	1.387	0.397	0.426	0.142
200	30	0.834	0.776	0.338	0.286	1.396	0.362	0.420	0.141
300	30	0.839	0.779	0.324	0.279	1.408	0.311	0.426	0.142
500	30	0.840	0.779	0.300	0.256	1.414	0.257	0.425	0.143
1,000	30	0.838	0.772	0.286	0.237	1.404	0.211	0.425	0.142
100	40	0.823	0.745	0.358	0.301	1.342	0.448	0.425	0.137
150	40	0.832	0.751	0.343	0.285	1.382	0.397	0.428	0.137
200	40	0.821	0.749	0.330	0.287	1.374	0.365	0.424	0.137
300	40	0.823	0.759	0.314	0.271	1.383	0.302	0.423	0.137
500	40	0.836	0.756	0.293	0.253	1.416	0.260	0.425	0.138
1,000	40	0.824	0.748	0.272	0.224	1.401	0.210	0.424	0.138

Patterns of bias also reflected this trend (Table 3). Similarly, the 3PL model was increasingly negatively biased in discrimination estimates with increasing sample size (e.g., −0.090 at *N* = 500, *I* = 6), whereas the bias for the 2PLE was small and relatively constant across conditions (e.g., −0.028 at *N* = 500, *I* = 6). These findings are consistent with the fact that when heterogeneous guessing behavior is forced into a fixed item-level lower asymptote, as in the 3PL, residual variance ends up being absorbed by the discrimination parameter, leading to systematic distortion. Because the 2PLE model accounts for guessing at the person level, it does bias the discrimination parameters in any way.

**TABLE 3:** Comparative performance of 3PL and 2PLE random models on bias estimates.

Sample size	Item count	Bias-θ-3PL	Bias-θ-2PLE R	Bias-a-3PL	Bias-a-2PLE R	Bias-b-3PL	Bias-b-2PLE R	Bias-c-3PL	Bias-g-2PLE R
100	6	0.018	−0.002	−0.022	−0.006	−1.367	−0.027	−0.387	0.001
150	6	0.021	0.009	−0.044	−0.006	−1.353	0.007	−0.387	0.001
200	6	−0.002	−0.012	−0.048	−0.001	−1.384	−0.015	−0.384	0.003
300	6	0.002	−0.003	−0.072	−0.032	−1.371	−0.024	−0.386	0.001
500	6	0.002	−0.002	−0.090	−0.028	−1.381	0.008	−0.386	0.000
1,000	6	0.011	0.009	−0.140	−0.074	−1.354	0.048	−0.386	0.000
100	10	0.027	−0.003	−0.060	−0.022	−1.367	−0.02	−0.390	−0.002
150	10	0.013	−0.009	−0.084	−0.025	−1.380	−0.02	−0.388	−0.001
200	10	0.011	−0.004	−0.109	−0.039	−1.373	0.002	−0.385	0.001
300	10	0.005	−0.006	−0.117	−0.055	−1.361	0.004	−0.386	0.000
500	10	0.001	−0.006	−0.127	−0.053	−1.367	0.032	−0.385	0.000
1,000	10	0.004	0.000	−0.130	−0.068	−1.379	0.033	−0.386	−0.001
100	20	0.038	−0.029	−0.054	−0.010	−−1.297	0.000	−0.385	−0.001
150	20	0.030	−0.013	−0.073	−0.016	−1.334	−0.012	−0.390	−0.004
200	20	0.052	0.019	−0.098	−0.027	−1.318	0.030	−0.386	0.000
300	20	0.023	0.000	−0.111	−0.028	−1.352	0.021	−0.385	0.000
500	20	0.024	0.009	−0.132	−0.045	−1.353	0.029	−0.388	−0.003
1,000	20	0.005	−0.002	−0.141	−0.072	−1.383	−0.004	−0.386	−0.002
100	30	0.080	−0.016	−0.069	−0.021	−1.245	0.027	−0.385	0.001
150	30	0.057	−0.005	−0.093	−0.024	−1.303	0.012	−0.388	−0.001
200	30	0.044	−0.009	−0.119	−0.032	−1.315	0.025	−0.383	0.001
300	30	0.034	−0.004	−0.121	−0.032	−1.338	0.018	−0.387	−0.003
500	30	0.015	−0.006	−0.131	−0.040	−1.348	0.013	−0.386	−0.002
1,000	30	0.016	0.005	−0.136	−0.060	−1.344	0.025	−0.386	−0.003
100	40	0.125	−0.004	−0.076	−0.012	−1.242	−0.002	−0.387	−0.006
150	40	0.106	0.020	−0.093	−0.014	−1.288	0.013	−0.390	−0.005
200	40	0.062	−0.006	−0.104	−0.023	−1.289	0.026	−0.385	−0.001
300	40	0.040	−0.006	−0.122	−0.029	−1.315	0.026	−0.385	−0.001
500	40	0.034	0.007	−0.124	−0.027	−1.346	0.010	−0.387	−0.003
1,000	40	0.015	0.001	−0.137	−0.053	−1.341	0.021	−0.385	−0.004

Bias values represent mean posterior bias across replications. Values closer to zero indicate better parameter recovery. R denotes the correctly specified 2PLE random-effects model.

### Item difficulty (b)

**FIGURE 3:**
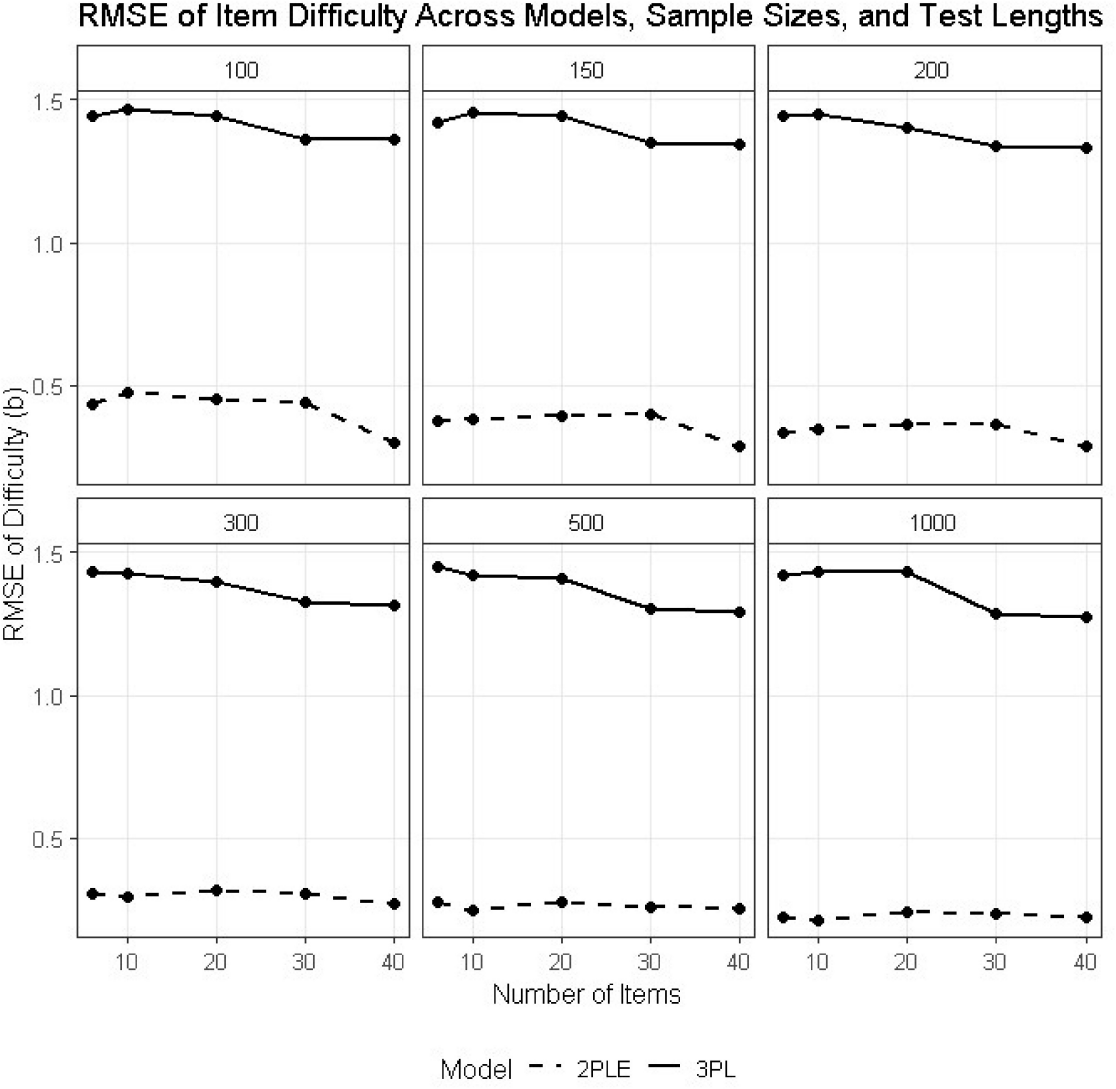
RMSE estimates for 3PL and 2PLE random models across simulation conditions for the difficulty parameter.

Interestingly, more pronounced differences emerged for the item difficulty parameters. Across conditions, the 3PL model produced large RMSE values for *b_i_*, oftentimes exceeding 1.40, with substantial negative bias (see Tables 2,3). For instance, at *N* = 500 and *I* = 6, the RMSE estimate for difficulty was 1.446 under the 3PL model, with a corresponding bias of −1.381. Similar patterns were observed across all test lengths (Figure 3).

In contrast, the 2PLE model yielded dramatically lower RMSE values for item difficulty (e.g., 0.274 at *N* = 500, *I* = 6) and bias values that were near zero or slightly positive (e.g., 0.008 at *N* = 500, *I* = 6). This pattern is theoretically consistent with the simulation design: when guessing varies across individuals but is constrained to be constant at the item level, as in the 3PL, item difficulty absorbs unexplained response variability, resulting in systematic underestimation. Allowing guessing to vary at the person level resolves this confounding and produces substantially more accurate and stable difficulty estimates.

### Guessing/lower-asymptote parameters (c or g)

Differences in the recovery of guessing-related parameters were similarly large. The 3PL model’s item-level pseudo-guessing parameter *c_i_* showed consistently high RMSE values (approximately 0.42 across all conditions) and large negative bias (approximately −0.38; Tables 2,3). In contrast, the 2PLE model’s person-level guessing probability *g_n_* was recovered more accurately with RMSE values for *g_n_* ranging between 0.14 and 0.17 across conditions. Bias estimates were consistently around zero for all sample sizes and test lengths. When heterogeneous guessing tendencies are forced into a single item-level constant, as in the 3PL, systematic underestimation appears to be unavoidable.

### Latent ability (θ)

**FIGURE 4:**
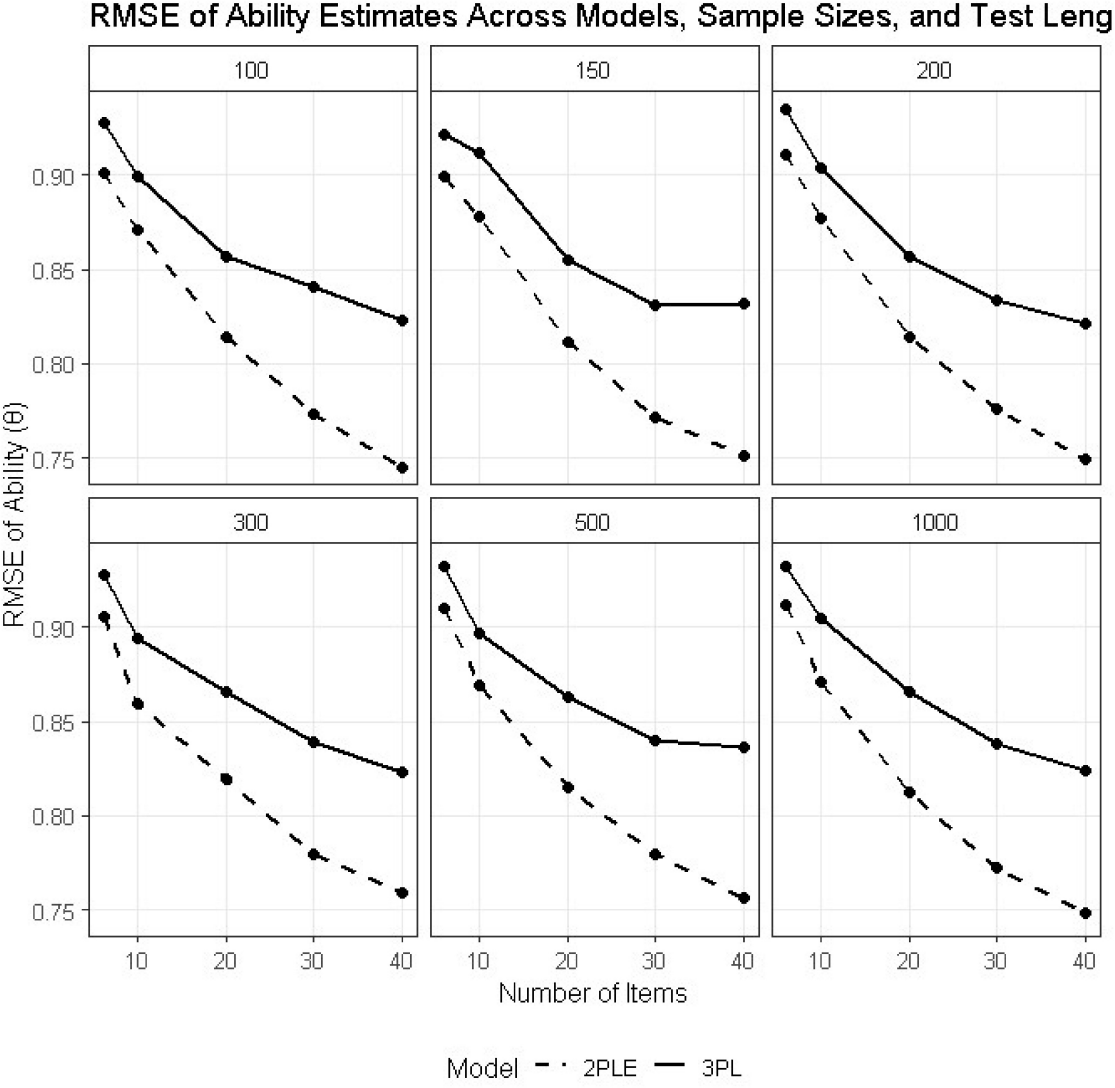
RMSE estimates for 3PL and 2PLE random models across simulation conditions for Theta.

Recovery of person ability also favored the 2PLE model across all conditions (Tables 2,3). In the smallest design condition (*N* = 100, *I* = 6), RMSE for ability decreased from 0.928 under the 3PL to 0.901 under the 2PLE model. Both models showed improved recovery with increasing sample size and test length; however, the relative advantage of the 2PLE model remained stable and, in some cases, increased slightly for longer tests (Figure 4).

Bias estimates for the 2PLE model were consistently close to zero across all conditions (e.g., −0.002 at *N* = 100, *I* = 6), whereas the 3PL model exhibited small but systematic bias (e.g., 0.018 at *N* = 100, *I* = 6). These results indicate that allowing guessing to vary across individuals improves the recovery of latent ability when heterogeneous guessing behavior is present, even though ability itself was generated independently of guessing propensity.

### Model fit and predictive accuracy

Model comparisons were conducted using LOOIC and WAIC, with lower values indicating better expected out-of-sample predictive accuracy. Differences were computed as ΔLOOIC = LOOIC(2PLE) − LOOIC(3PL), such that negative values favor the 2PLE model. To assess the reliability of these differences, we report the standard error of ΔLOOIC, computed from pointwise log-likelihood differences using Pareto-smoothed importance sampling as implemented in the loo package. Following established recommendations, differences exceeding approximately twice their standard error were interpreted as providing meaningful evidence in favor of one model over the other (Table 4). Both indices approximate out-of-sample predictive accuracy, with lower values indicating better performance. Pareto-k diagnostics indicated that PSIS-LOO estimates were reliable across all conditions, with mean k values well below the critical threshold of 0.7 and virtually no observations exceeding this cutoff (Supplementary Appendix 3). Model-averaging weights using stacking and pseudo-BMA showed preference for the 2PLE model across all test lengths (see Supplementary Appendix 4).

**TABLE 4:** Comparative performance of 3PL and 2PLE random models on global fit statistics.

Sample size	Item count	LOOIC-3PL	LOOIC-2PLE R	WAIC-3PL	WAIC-2PLE R	PPC
100	6	652.252	650.910	650.631	650.004	0.740
150	6	960.904	958.775	958.849	957.548	0.747
200	6	1298.88	1296.955	1296.287	1295.28	0.738
300	6	1896.088	1894.555	1893.035	1892.307	0.752
500	6	3232.025	3229.351	3227.79	3225.52	0.741
1,000	6	6445.479	6443.704	6439.532	6436.573	0.741
100	10	1066.759	1064.528	1065.005	1063.562	0.748
150	10	1608.11	1603.402	1605.9	1601.988	0.741
200	10	2116.649	2112.614	2114.089	2110.844	0.745
300	10	3174.567	3170.540	3171.362	3167.964	0.743
500	10	5326.091	5323.295	5322.11	5319.27	0.742
1,000	10	10701.841	10697.912	10695.997	10690.325	0.737
100	20	2116.586	2110.543	2114.679	2109.467	0.738
150	20	3143.56	3135.825	3141.32	3134.373	0.742
200	20	4188.924	4178.846	4186.396	4176.997	0.743
300	20	6323.656	6314.244	6320.664	6311.653	0.741
500	20	10515.374	10502.945	10511.512	10498.865	0.739
1,000	20	20892.339	20881.040	20886.799	20873.771	0.741
100	30	3136.151	3122.592	3134.257	3121.482	0.741
150	30	4660.24	4644.878	4658.097	4643.421	0.746
200	30	6302.241	6286.086	6299.848	6284.215	0.739
300	30	9475.455	9455.393	9472.638	9452.854	0.735
500	30	15573.169	15551.779	15569.668	15547.876	0.741
1,000	30	31088.561	31061.544	31083.432	31054.791	0.743
100	40	4205.957	4186.395	4204.071	4185.192	0.738
150	40	6229.544	6208.827	6227.436	6207.294	0.743
200	40	8312.107	8285.279	8309.78	8283.378	0.740
300	40	12468.4	12439.534	12465.724	12436.954	0.740
500	40	20675.323	20640.691	20672.021	20636.879	0.741
1,000	40	41389.138	41349.500	41384.262	41342.993	0.740

Lower values of LOOIC and WAIC indicate better out-of-sample predictive performance. Posterior predictive *p*-values (PPC) close to 0.50 indicate adequate model fit.

The magnitude of the effects was relatively small in absolute terms, typically ranging from 1 to 20 points; however, it was consistent across groups. For instance, in the *N* = 100, *I* = 6 condition, LOOIC was reduced from 652.25 under the 3PL to 650.91 under the 2PLE model, and WAIC decreased from 650.63 to 650.00. In the largest sample condition (*N* = 1000, *I* = 6), the 2PLE model also yielded lower LOOIC (6443.70 vs. 6445.48) and WAIC (6436.57 vs. 6439.53). This trend was found to hold also for tests of length 10, 20, 30, and 40 items.

The stability of such differences suggests that the superior performance of the 2PLE is not contingent on specific sample sizes or test lengths. Rather, it is an inherent structural advantage due to modeling guessing as a person-level function rather than a fixed item-level constant. Such flexibility enables the 2PLE to model response styles across a wider range of ability and hence increase the out-of-sample predictive performance.

### Posterior predictive checks

**FIGURE 5:**
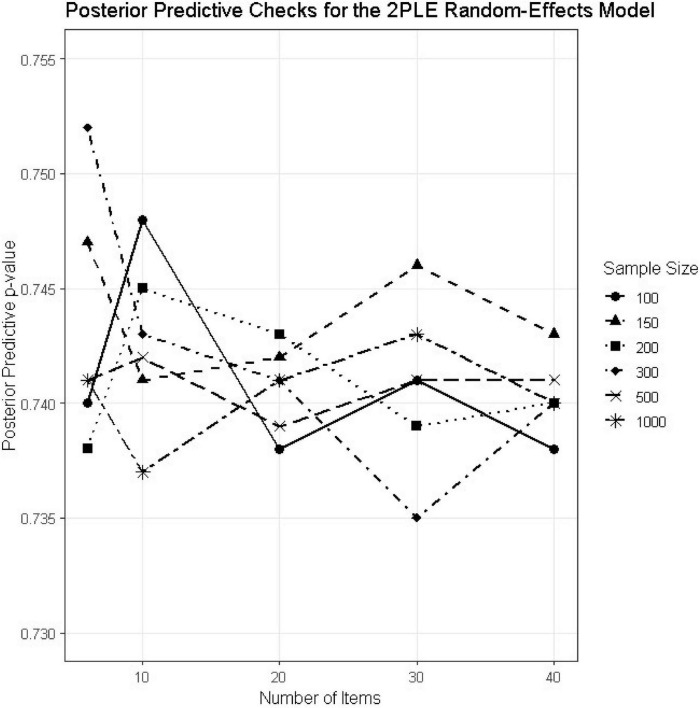
PPC estimates for the 2PLE random models across simulation conditions.

Posterior predictive *p*-values (PPCs) were computed for all simulation conditions to evaluate model adequacy (Table 4). Absolute model adequacy was evaluated by posterior predictive p-values (PPCs) for all 30 simulation conditions (Table 4). Posterior predictive p-values were tightly concentrated around 0.74 under all simulation conditions, which meant that there was no systematic bias between observed and reproduced data (Figure 5). While PPC values around 0.50 frequently get highlighted as perfect, PPC over the range 0.70–0.80 are typical of correctly specified hierarchical models and correspond to conservative test statistics rather than model misspecification. The lack of extreme PPC values and steady trends over N or test length constitute compelling evidence for model appropriateness. Together with the smooth monotonic improvement in RMSE and the invariant direction of ΔLOOIC across conditions, these results indicate that the 2PLE model performs robustly across a wide range of realistic testing scenarios.

### Robustness across design conditions

Taken together, the results demonstrate that the advantages of the 2PLE model are robust across a wide range of realistic testing scenarios. RMSE, bias, LOOIC, WAIC, and PPC values all varied smoothly with increases in sample size and test length and consistently favored the 2PLE model. No reversals or instabilities were observed for any evaluation metric.

### Real data example using PIRLS 2019

Models were fitted on data of a reading comprehension scale from PIRLS 2019 and the country Saudi Arabia. PIRLS is an international assessment, which was developed to assess the reading comprehension of fourth-grade students by measuring their ability to construct meaning from written text in literary and informational domains. Items were contextualized within narrative and information passages about animal behavior and survival—a typical PIRLS theme designed to be engaging for young readers, while tapping different levels of comprehension. Each item was scored as correct (1) or incorrect (0) and involved students in responding to explicitly stated information, making simple inferences, or combining local cues with prior knowledge. In the present evaluation participants were 413 4th graders, with 207 being females (50.1%) and 206 males (49.9%). Among them, 357 (86.4%) were of Arabic origin and 56 (13.6%) were international students.

#### Model fit and predictive comparisons

The reading comprehension subscale was used in the comparison between the 2PLE and 3PL models using as criteria the Widely Applicable Information Criterion (WAIC) and Pareto-smoothed importance sampling leave-one-out cross-validation (PSIS-LOOIC). Across analyses, the two models demonstrated remarkably similar predictive performance [LOOIC_2PLE_ = 2889.604, S.E._2PLE_ = 43.263; LOOIC_3PL_ = 2888.117, S.E._3PL_ = 43.597; WAIC_2PLE_ = 2878.797, S.E_2PLE_. = 42.986; WAIC_3PL_ = 2889.029, S.E._3PL_ = 43.449]. Differences in both WAIC and LOOIC were small in absolute magnitude (Δ < 2 points) and trivial relative to the associated standard errors, indicating that neither model achieved a substantively meaningful advantage in expected out-of-sample prediction.

#### Person-level versus item-level guessing: substantive considerations

Although differences between the 2PLE and 3PL models were marginal, models differed fundamentally in the location of guessing behavior. Within the 3PL model, guessing is modeled as an item-specific lower asymptote that indirectly accounts for unexpected correct responses from low-ability examinees based on characteristics of individual items (e.g., distractor quality or cueing). In 2PLE, however, guessing is represented as a latent person-level variable that accounts for stable individual differences in the likelihood of guessing across items.

The findings from the 2PLE model showed pronounced individual differences in the person-level guessing probabilities with posterior mean estimates indicating that guessing was not restricted to few items but rather was an important response process at the examinee level. Interestingly, the person-level guessing variance was estimated without compromising overall predictive accuracy relative to the 3PL model. Thereby, the inclusion of a latent guessing trait did not result in overfitting or deterioration of out-of-sample performance evident by similar LOOIC and WAIC values across models.

#### Rationale for preferring the 2PLE model against the 3PL model

**FIGURE 6:**
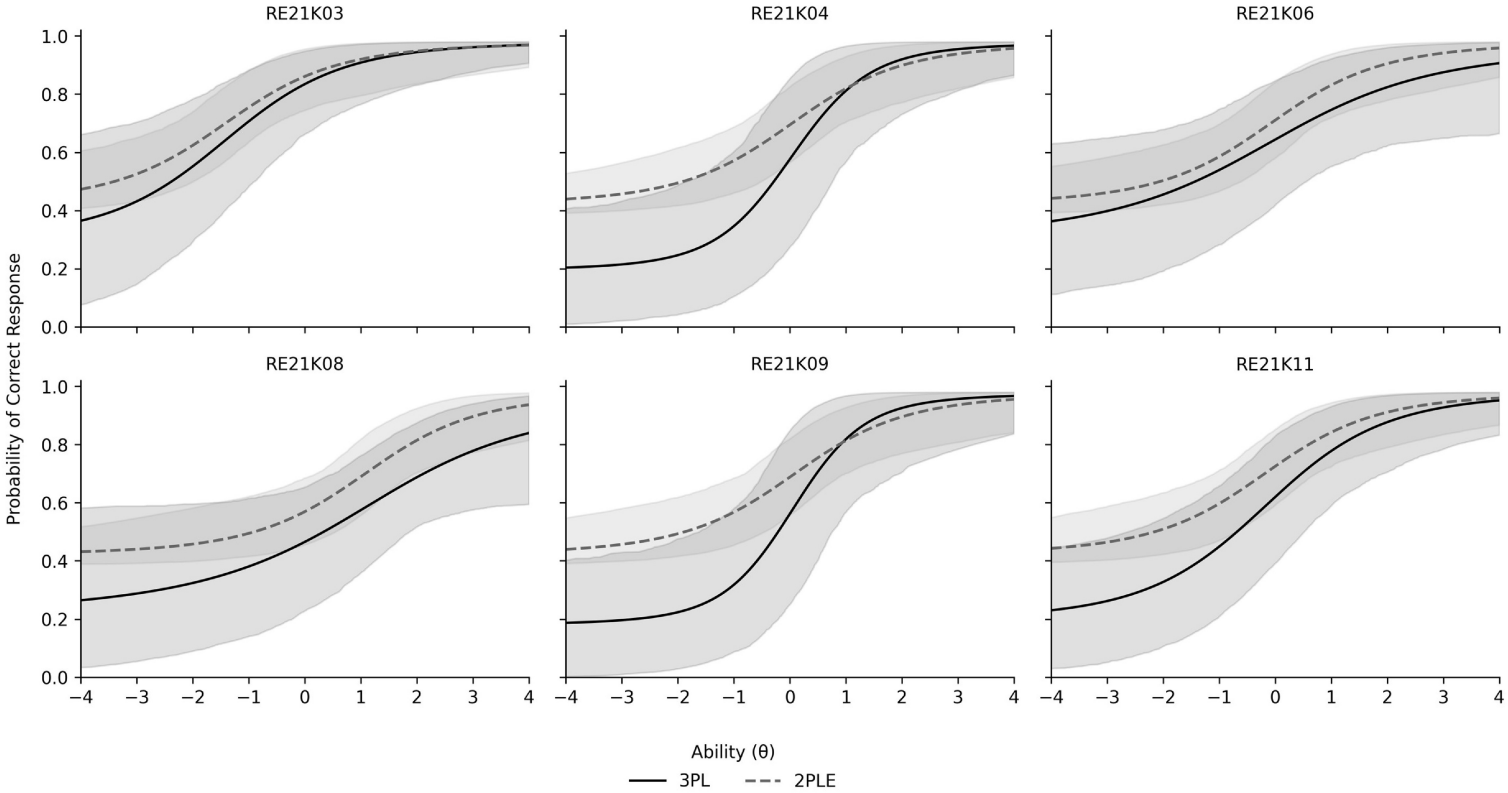
Item characteristic curves and credible intervals for PIRLS reading comprehension items contrasting the 2PLE with the 3PL models.

As the predictive fit was empirically equivalent between model comparisons, substantive interpretability and conceptual alignment to the measurement construct, as opposed to incremental changes in information criterion, were used for model selection. Reading comprehension tests like PIRLS do not simply evaluate reading, but individual response tendencies to read complex texts in specific timeframes. Within this context, guessing can reasonably be construed as a person- level response tendency (e.g., with respect to item-specific thresholds as opposed to category-boundaries) that represents inter-individual variation in test- taking strategy, motivation, risk willingness or partial understanding rather than merely an item-level artifact. The 2PLE model directly parameterizes this individual-difference process by estimating posterior distributions of person-level guessing probabilities. Indeed, such an attribute allows the possibility of psychometrically meaningful inferences which cannot be provided within the 3PL model. Thus, the 2PLE model offers a conceptually coherent framework for modeling guessing behavior in PIRLS reading comprehension data. Figure 6 displays the ICCs of the reading comprehension items along with their credible intervals which are notable much more narrow in the case of the 2PLE model.

## Discussion

The present study examined the consequences of modeling guessing behavior as a latent person-level trait rather than as a fixed item-level constant. We compared the traditional 3PL model and a corresponding variation of the Two-Parameter Logistic Extension with person-specific guessing (2PLE random-effects model) using an extensive Monte Carlo study. Across various sample sizes and test lengths, the results tended to prefer 2PLE model in parameter recovery, bias reduction, and predictive fit. Collectively, these findings demonstrate that when guessing behavior varies systematically across individuals, constraining guessing to the item level induces substantial and predictable distortions in item and ability estimation. These are consistent with prior work documenting limitations of item-level guessing parameters in the presence of heterogeneous examinee behavior which suggest that fixed guessing parameters can distort item difficulty estimates and inflate discrimination when guessing varies across individuals (Reise and Waller, 2009; van der Linden, 2007). Our results corroborate these findings by demonstrating systematic bias in difficulty estimates under the 3PL model when person-level guessing heterogeneity is present. Beyond confirming prior concerns, the present study extends the literature by formally modeling guessing as a person-level latent trait rather than as an item-level nuisance parameter. Whereas earlier approaches either fixed guessing at the item level or treated aberrant responding as a post hoc diagnostic problem, the 2PLE model integrates guessing directly into the measurement model, allowing individual differences in guessing propensity to be estimated simultaneously with ability.

### Theoretical implications

Psychometrically, estimating guessing as an individual-level construct is congruent with classical and contemporary measurement models. Guessing is usually subsumed under random error variance in Classical Test Theory with the implicit assumption that its effects are purely unchanging and unsystematic. These results therefore question the assumption that guessing is unsystematic and represents measurement error. From the standpoint of latent trait theory this perspective aligns naturally with the representation of examinee-specific tendencies, such as engagement, risk-taking, or response strategies, as latent variables that interact with ability to produce observed responses.

A central theoretical contribution of this is that guessing should not be considered only an item characteristic or a nuisance parameter. Rather, the simulation data are consistent with guessing being conceptually represented as an individual-level unobservable trait that mediates interaction between ability and response. This view is consistent with research on person-fit and aberrant response behavior which suggests that behaviors such as rapid guessing, disengagement, and impulsive responding often reflect stable individual tendencies rather than random noise (Karabatsos, 2003; Wise and Smith, 2011). From this perspective, the failure of the 3PL model is not only one of estimation but also a psychometric one in that guessing is likely not an item characteristic but a person trait.

Importantly, the results are critical in understanding the misspecification caused by applying heterogeneous guessing at item-level. If guessing varies between persons but is constrained to be the same within items, as it is in the 3PL model, residual response variability is switched systematically to other parameters such as item difficulty and discrimination. This mechanism was evident with large amounts of negative bias and inflated RMSE in the difficulty parameters under the 3PL model across all simulation conditions. Through moving the guessing random variable from items to persons, the 2PLE model separates out these two sources of uncertainty and provides parameter estimates that align more closely with true examinee behavior.

### Methodological implications

From a methodological standpoint, this study provides compelling evidence that person-level guessing models can substantially improve both local parameter recovery and global predictive performance. This effect was true across all 30 simulation conditions with the 2PLE model being associated with lower RMSE and bias for item discrimination, item difficulty, guessing-related parameters, and latent ability. Interestingly the effects were consistent even with small sample sizes and test lengths. Differences in global fit (LOOIC and WAIC) were modest in magnitude but consistent in that the 2PLE model outperformed the 3PL model across all conditions. In Monte Carlo simulations, consistent directional advantages across conditions add strength to the superiority of one methodology over another.

Importantly, the benefits of using the 2PLE model were most pronounced when there was substantial person-level guessing heterogeneity—conditions where unmodeled response behavior is most susceptible to biasing parameter estimates. Under these conditions, the 3PL model was consistently biased toward item difficulty and overly inflated uncertainty in ability estimates whereas the bias of the 2PLE model did not grow. These results also indicate that it is especially important to account for examinee engagement in realistic testing conditions and where we should not assume person-level guessing to be equal across all individuals.

### Implications for ability estimation

Enhanced recovery of latent ability under the 2PLE model has serious consequences for score interpretation and validity. Although ability and guessing tendencies were independently simulated, the impact of heterogeneous guessing behavior on ability estimation was observed under the 3PL model misspecification. Allowing guessing to vary at the person level, the 2PLE model also improved bias and RMSE in ability estimates across all conditions. This result highlights that accurate ability estimation is not simply a matter of modeling ability alone, but also involves representing the ancillary response processes that produce performance on observed tests.

In practice, these findings indicate that inadequate consideration of individual differences in response to guessing may result in biased proficiency estimates, particularly for examinees with relatively low ability or who demonstrate less attentive modes of responding. In contrast, person-level guessing models provide a principled means to tease apart true ability from response strategies and thus strengthen construct validity.

### Applied interpretation of guessing models in PIRLS reading comprehension

The differentiation between the 2PLE and 3PL models has implications for large-scale reading comprehension assessments like PIRLS, where scores are utilized to make inferences at the policy level. Items on the PIRLS reading assessment are constructed to measure comprehension processes from literal recall to inferential reading and integration. In these assessments, false positives and negatives by weaker learners can occur not just through an absence of understanding but for strategic reasons, partial grasp, disengagement or educated guessing in the face of uncertainty. From an applied measurement perspective, the central question is therefore not merely which model yields marginally better global fit, but where the model locates the source of unexpected correct responses. The 3PL model attributes these relations directly to item-specific characteristics, assuming guessing to be a function of distractor quality or the extent of cueing from item presentation. In contrast, the 2PLE model treats guessing as person-level response style by which individual differences in test-taking behavior are more directly modeled within reading ability. In comparing such models in practice, then attention needs to swift from information criteria to which model is more substantively interpretable for the latent construct measured.

In the current analyses, differences in relative fit between the two models were negligible and we believe that the 2PLE model is more interpretable and thus more optimal. The advantage of the 2PLE model over alternative item response models in PIRLS lies in its ability to derive estimation on differences of guessing propensity without reliance on substantive understanding of student response behavior, which is crucial for valid interpretation

### Practical implications

The results have straightforward implications for both educational and psychological measurement, particularly in situations where the engagingness of examinees to test items may vary. In standardized testing, professional licensure exams and other low-stakes assessment environments heterogeneous guessing is likely common and may introduce threats to validity due to guessing. The 2PLE model offers a more flexible and theoretically motivated approach to this problem by treating guessing as a person level property rather than an item level attribute.

The relevance of the 2PLE model is not limited to better parameter recovery, however. Person-level guessing estimates might also be of use for detecting when low-ability examinees are responding in ways that deviate from human models of answering behavior (e.g., by guessing very quickly or not at all) in test security contexts to supplement currently available methods. Detaching the guessing effect from ability in computerized adaptive testing can be beneficial for initial-item selection and ability estimation, especially when little information is offered. More generally, by controlling for the confounding of item characteristics and examinee behavior, the 2PLE model enables accurate measurement with fairer inferences from scores in educational and psychological testing.

In addition, the person-level guessing trait as estimated by the 2PLE may have clinical utility beyond enhancing parameter estimation. While the current investigation did not test content interpretations of this parameter, it likely represents aspects like test-taking engagement, impulsivity or response caution. As with other person-fit indices and response-time–based measures, it could augment the utility of existing measures by providing a unique perspective on examinee behavior without needing to rely on additional sources of data.

### Limitations and future directions

The present study is subject to several limitations. First, the current formulation models guessing solely as a person-level latent trait. An important extension would be to develop person × item guessing models, in which individual guessing propensities interact with item features such as format, cognitive demand, or number of response options. Such models could further disentangle strategic guessing from item-specific affordances. Second, the guessing as modeled by the present instantiation is only through person-level latent trait. An important extension will be to further model response bias in a person × item format (guessing models) in which one’s guessing propensity is influenced by characteristics of the items, by their level of cognitive demand or number of alternatives that might be guessed. Such models might serve to disentangle strategic guessing from item-specific affordances. Finally, future studies should expand the model to other measurement settings such as low-stakes testing, large-scale assessment and diagnostic testing venues, or adopt joint modeling outcomes that incorporate RTs or engagement. Taken together, these extensions would help to explicate the function of person-level guessing in educational and psychological measurement, and expand the utility of this model.

## Conclusion

The current simulation study has shown that modeling guessing as a person-specific latent trait provides large measurement advantages when some individuals guess while others do not. In contrast to the standard 3PL model, the 2PLE random-effects model produced superior recovery of item and person parameters, less bias, and consistently better predictive fit over a wide range of test circumstances. These benefits are not simply a consequence of model nonlinearity but result from more accurate encoding of the process generating responses. Collectively, these results suggest that guessing is not simply a nuisance or deviant behavior to be screened out during the *post-hoc* analysis but is an important part of the responding process that can—and should—be specifically modeled when individual differences in test-taking are present.

## Data Availability

Publicly available datasets were analyzed in this study. This data can be found at: https://www.iea.nl/data-tools/repository/pirls.
