# Supplementary material for: Modeling person guessing as a random effect: a Bayesian approach of the two-parameter logistic model
Source: Front Psychol. 2026 Feb 16;17:1678086. doi: 10.3389/fpsyg.2026.1678086 (PMC12950595; doi:10.3389/fpsyg.2026.1678086)
Supplement: Supplementary file 1 [file Table_1.docx]

**Supplementary Material**

**Appendix A**

*Stan Code for 2PLE Model*

data {

int<lower=1> N; // number of persons

int<lower=1> I; // number of items

array[N, I] int<lower=0, upper=1> Y; // response matrix

real<lower=0, upper=1> u; // fixed upper asymptote (e.g., 0.98)

}

parameters {

// -------------------------------------------------

// Non-centered parameterization for person ability

// -------------------------------------------------

real mu_theta; // population mean ability

real<lower=0> sigma_theta; // population SD of ability

vector[N] z_theta; // standardized ability (NCP)

// -------------------------------------------------

// Non-centered parameterization for person guessing

// -------------------------------------------------

real mu_delta; // population mean guessing propensity

real<lower=0> sigma_delta; // population SD of guessing propensity

vector[N] z_delta; // standardized guessing trait (NCP)

// -------------------------------------------------

// Item parameters

// -------------------------------------------------

vector<lower=0>[I] a; // item discrimination

vector[I] b; // item difficulty

}

transformed parameters {

// -------------------------------------------------

// Non-centered transformations

// -------------------------------------------------

vector[N] theta = mu_theta + sigma_theta * z_theta;

vector[N] delta = mu_delta + sigma_delta * z_delta;

// -------------------------------------------------

// Person-level guessing probabilities

// -------------------------------------------------

vector[N] g;

for (n in 1:N)

g[n] = inv_logit(delta[n]);

}

model {

// -------------------------------------------------

// Priors for non-centered latent variables

// -------------------------------------------------

z_theta ~ normal(0, 1);

z_delta ~ normal(0, 1);

// -------------------------------------------------

// Hyperpriors

// -------------------------------------------------

mu_theta ~ normal(0, 1);

sigma_theta ~ normal(0, 1);

mu_delta ~ normal(0, 1);

sigma_delta ~ normal(0, 1);

// -------------------------------------------------

// Item parameter priors

// -------------------------------------------------

a ~ lognormal(0, 0.5);

b ~ normal(0, 1);

// -------------------------------------------------

// Likelihood: 2PLE random-effects model

// -------------------------------------------------

for (n in 1:N) {

for (i in 1:I) {

real p;

p = g[n] + (u - g[n]) * inv_logit(a[i] * (theta[n] - b[i]));

Y[n, i] ~ bernoulli(p);

}

}

}

generated quantities {

// -------------------------------------------------

// Log-likelihood for model comparison (LOO / WAIC)

// -------------------------------------------------

array[N, I] real log_lik;

for (n in 1:N) {

for (i in 1:I) {

real p;

p = g[n] + (u - g[n]) * inv_logit(a[i] * (theta[n] - b[i]));

log_lik[n, i] = bernoulli_lpmf(Y[n, i] | p);

}

}

}

*Stan Code for 3PL Model*

# data {

# int<lower=1> N;

# int<lower=1> I;

# array[N, I] int<lower=0, upper=1> Y;

# real<lower=0, upper=1> u; // fixed upper asymptote (e.g., 0.98)

# }

# parameters {

# vector[N] theta;

# vector<lower=0>[I] a;

# vector[I] b;

# vector<lower=0, upper=0.5>[I] c;

# }

# model {

# // Priors

# a ~ lognormal(0, 0.5);

# b ~ normal(0, 2);

# c ~ beta(2, 17);

# theta ~ normal(0, 1);

# // Likelihood

# for (n in 1:N)

# for (i in 1:I) {

# real eta = c[i] + (u - c[i]) * inv_logit(a[i] * (theta[n] - b[i]));

# eta = fmin(fmax(eta, 1e-9), 1 - 1e-9);

# Y[n, i] ~ bernoulli(eta);

# }

# }

# generated quantities {

# array[N, I] real log_lik;

# for (n in 1:N)

# for (i in 1:I) {

# real eta = c[i] + (u - c[i]) * inv_logit(a[i] * (theta[n] - b[i]));

# eta = fmin(fmax(eta, 1e-9), 1 - 1e-9);

# log_lik[n, i] = bernoulli_lpmf(Y[n, i] | eta);

# }

# }

# Appendix B1. Bayesian Estimation Settings and Convergence Diagnostics

MCMC Settings and Convergence Diagnostics

| **Model** | **Chains** | **Iterations** | **Warm-up** | **Sampler** | **Max R̂** | **Min ESS (bulk)** | **Divergences** |
| --- | --- | --- | --- | --- | --- | --- | --- |
| 3PL | 4 | 2000 | 1000 | NUTS | 1.02 | 320 | 0 |
| 2PLE Random | 4 | 2000 | 1000 | NUTS | 1.02 | 410 | 0 |

Note. All models converged satisfactorily with no divergent transitions.

# Appendix C1. Pareto-k Diagnostics for LOOIC Reliability

Pareto-k Diagnostics Across Simulation Conditions

| **Sample Size** | **Item Count** | **Mean k (3PL)** | **Mean k (2PLE-R)** | **% k > 0.7 (3PL)** | **% k > 0.7 (2PLE-R)** |
| --- | --- | --- | --- | --- | --- |
| 100 | 6 | 0.27 | 0.17 | 0.09% | 0.00% |
| 100 | 10 | 0.24 | 0.16 | 0.07% | 0.00% |
| 100 | 20 | 0.21 | 0.15 | 0.04% | 0.00% |
| 100 | 30 | 0.19 | 0.14 | 0.02% | 0.00% |
| 100 | 40 | 0.18 | 0.13 | 0.01% | 0.00% |
| 300 | 6 | 0.22 | 0.15 | 0.05% | 0.00% |
| 300 | 10 | 0.19 | 0.14 | 0.03% | 0.00% |
| 300 | 20 | 0.17 | 0.13 | 0.01% | 0.00% |
| 300 | 30 | 0.15 | 0.12 | 0.00% | 0.00% |
| 300 | 40 | 0.14 | 0.11 | 0.00% | 0.00% |
| 1000 | 6 | 0.15 | 0.12 | 0.01% | 0.00% |
| 1000 | 10 | 0.13 | 0.11 | 0.00% | 0.00% |
| 1000 | 20 | 0.11 | 0.10 | 0.00% | 0.00% |
| 1000 | 30 | 0.10 | 0.10 | 0.00% | 0.00% |
| 1000 | 40 | 0.10 | 0.10 | 0.00% | 0.00% |

Note. Lower Pareto-k values indicate more reliable LOOIC estimates. All conditions fall well below the critical threshold (k = 0.7).

# Appendix D1. Model Weights from Bayesian Model Averaging

*Average Predictive Model Weights by Test Length*

| **Item Count** | **Stacking Weight (3PL)** | **Stacking Weight (2PLE-R)** | **Pseudo-BMA (3PL)** | **Pseudo-BMA (2PLE-R)** |
| --- | --- | --- | --- | --- |
| 6 | 0.47 | 0.53 | 0.49 | 0.51 |
| 10 | 0.44 | 0.56 | 0.45 | 0.55 |
| 20 | 0.40 | 0.60 | 0.41 | 0.59 |
| 30 | 0.36 | 0.64 | 0.35 | 0.65 |
| 40 | 0.32 | 0.68 | 0.30 | 0.70 |

Note. Higher weights indicate superior expected predictive performance. Weights increasingly favor the 2PLE-R model as test length increases.
